# Fine-tuning CRISPR/Cas9 gene editing in common bean (*Phaseolus vulgaris* L.) using a hairy root transformation system and *in silico* prediction models

**DOI:** 10.3389/fpls.2023.1233418

**Published:** 2023-10-20

**Authors:** Ramon de Koning, Hana Daryanavard, Joyce Garmyn, Raphaël Kiekens, Mary Esther Muyoka Toili, Geert Angenon

**Affiliations:** ^1^ Research Group Plant Genetics, Department of Bioengineering Sciences, Vrije Universiteit Brussel, Brussels, Belgium; ^2^ Department of Horticulture, Jomo Kenyatta University of Agriculture and Technology, Nairobi, Kenya

**Keywords:** *P. vulgaris*, legumes, Raffinose family oligosaccharides (RFO), transformation, *R. rhizogenes*, gene editing, CRISPR, inDelphi

## Abstract

The stable transformation of common bean is a challenging and time-consuming process. Although CRISPR/Cas9 has revolutionized gene editing with its high efficiency and specificity, the performance of the system can be affected by multiple factors, such as sgRNA specificity and effectiveness, and the choice of promoter used to drive Cas9 expression. The use of a hairy root transformation system to initially check the efficiency of sgRNAs and the impact of different promoters could speed up this process and increase the chances of success. We initially tested three different transformation methods to induce hairy roots and selected a preferred method suitable for a variety of different common bean genotypes. This method involved inoculating a severed radicle with *Rhizobium rhizogenes* K599 and was fast, had a high transformation frequency of 42-48%, and resulted in numerous hairy roots. This method was further used for the transformation of explants using *R. rhizogenes* harboring different CRISPR/Cas9 constructs and evaluated the on-target activity of sgRNAs targeting raffinose family oligosaccharides biosynthetic genes and the impact of different promoters driving Cas9 on the gene editing efficiency. Additionally, we evaluated the reliability of the *in silico* tools, CRISPOR, CRISPR RGEN, and inDelphi to predict the sgRNA efficiencies and resulting mutations. Our results showed that the hairy root transformation system allows for rapid evaluation of multiple sgRNAs and promoters. We also identified several highly efficient sgRNAs that induced frameshift mutations at rates of up to 70% when a parsley ubiquitin promoter was driving Cas9 expression, providing valuable information for the selection of the most effective sgRNAs and promoters for future transformation experiments. Although most of the computational models used to predict the sgRNA efficiency did not match the *in planta* results, the Lindel model proved to be the most reliable for *P. vulgaris*, accurately predicting the sgRNA efficiency and the type of induced mutation in most hairy roots. Furthermore, the inDelphi algorithm could correctly predict deletions and single nucleotide insertions resulting from DNA double-strand breaks in common bean. These results offer promising implications for enhancing precise editing in plants because they provide the possibility of predicting repair outcomes.

## 1 Introduction

Common bean (*Phaseolus vulgaris* L.) is a crucial crop in global agriculture, providing a significant source of dietary protein and essential nutrients for millions of people worldwide (Doria et al., 2012; Ganesan and Xu, 2017). Given its importance, there is an ongoing need for crop improvement strategies to enhance yield, nutritional quality, and stress tolerance, among other traits (Assefa et al., 2019). Recent advances in plant biotechnology, specifically the development of clustered regularly interspaced short palindromic repeat (CRISPR) and CRISPR associated protein 9 (Cas9) based genome-editing techniques, have opened new avenues for crop improvement that are faster, more precise, and cost-effective than traditional breeding methods (Mousavi-Derazmahalleh et al., 2019).

CRISPR/Cas9 is a powerful tool that facilitates targeted modifications to a plant’s genome by inducing double-strand breaks (DSBs) in DNA at specific locations, which are subsequently repaired through the plant’s inherent DNA repair mechanisms. Canonical non-homologous end-joining (C-NHEJ) is the primary mechanism for repairing DSBs in plants; however, this process is error-prone and can result in small deletions and insertions of a few base pairs (Puchta, 2005; Weterings and Chen, 2008). Alternatively, microhomology-mediated end joining (MMEJ), also referred to as alternative NHEJ (ALT-NHEJ), relies on microhomologies (1-8 nucleotides) near the DSB site, leading to small deletions (Seol et al., 2018; Van Vu et al., 2021). If the broken DNA ends have longer homologous sequences situated further from the DSB, single-strand annealing (SSA) may occur. Similar to MMEJ, SSA involves annealing homologous regions to bridge the DSB, followed by resection of overhanging sequences, gap filling, and ligation to repair the DSB, resulting in larger deletions (Bhargava et al., 2016). Additionally, plants can repair a DSB through homologous recombination (HR), a highly accurate repair mechanism that utilizes a homologous DNA sequence (typically a sister chromatid) to guide the repair of the DSB. Nonetheless, HR plays a minor role as a DSB repair system in plant somatic cells (Puchta, 2005; Weterings and Chen, 2008). All these repair processes can lead to mutations that result in gene knockouts within the plant (Ma et al., 2016; Zhang et al., 2021).

Gene editing in plants requires optimization at the species or genotype level (Rasheed et al., 2022; Son and Park, 2022). Common bean transformation remains challenging, i.a. because of the lengthy process, low efficiency, and genotype dependence (Hnatuszko-Konka et al., 2014). Furthermore, the efficiency of the CRISPR/Cas9 system can be affected by several factors, such as codon usage in the Cas9 encoding gene, the promoter driving Cas9 expression, and the editing efficiency and specificity of the single guide RNA (sgRNA) used to target the desired genomic locus (Ma et al., 2015; Liang et al., 2016; Ma et al., 2016; Li et al., 2022). These factors highlight the need for a rapid and efficient screening method to validate sgRNA efficiency in common bean before embarking on the more time-consuming stable transformation process. CRISPOR and RGEN are the only two bioinformatic programs currently available for designing sgRNAs in common bean, as they include the species’ reference genome in their database. However, these computational tools are mainly trained on datasets from human, mouse, and zebrafish cells, which limits their applicability to plant systems (Moreno-Mateos et al., 2015; Park et al., 2015; Doench et al., 2016; Chen et al., 2019; Li et al., 2022). Additionally, different Cas9/sgRNA delivery methods have been used in plants compared to animal systems, further complicating the translation of predicted sgRNA efficiencies to actual on-target activity *in planta*. One potential solution for rapidly assessing sgRNA efficiency in common bean is the use of the hairy root transformation system. Hairy root induction, mediated by *Rhizobium rhizogenes*, is a widely used model system for studying gene function and secondary metabolite biosynthesis in various plant species (Gutierrez-Valdes et al., 2020; Alamillo et al., 2023). Although hairy root transformation protocols have been established for common bean in the past, genotype specificity remains a challenge (Estrada-Navarrete et al., 2006; Colpaert et al., 2008; Khandual and Reddy, 2014; Voß et al., 2022).

In this study, we aimed to develop a rapid and efficient hairy root transformation system for common bean cv. CIAP7247F by testing three different methods of hairy root induction. Once a preferred method was identified, we used this system to assess the *in planta* efficiency of *in silico*-designed sgRNAs targeting genes from the raffinose family oligosaccharides (RFOs) metabolic pathway in common bean (de Koning et al., 2021; de Koning et al., 2023). Additionally, we tested two CRISPR vector constructs that differed in the promoter driving Cas9 expression. The first construct contained the cauliflower mosaic virus 35S promoter with a duplicated enhancer and an omega translational enhancer from tobacco mosaic virus (2x35S-Ω), while the second construct employed the *Petroselinum crispum* (Parsley) ubiquitin promoter (PcUbi). This allowed us to investigate the impact of these promoters on the CRISPR/Cas9 editing efficiency. Both promoters are widely used as gene regulators in eudicots and are considered strong promoters (Kay et al., 1987; Fauser et al., 2014; Bortesi and Fischer, 2015; Shan et al., 2020; Baloglu et al., 2022). By comparing the efficiency of sgRNAs and the promoters driving Cas9, we aimed to identify the most effective combination for use in stable transformation experiments targeting the RFO biosynthetic genes in common bean. RFOs are a group of sugars that act as antinutritional factors in food and feed for monogastric animals and humans, thus negatively affecting digestion and absorption of nutrients (Coon et al., 1990; Tomlin et al., 1991; Valentine et al., 2017). Therefore, reducing the RFO content in common bean could increase its nutritional value (Valentine et al., 2017). We also aimed to evaluate the reliability of *in silico* prediction tools for sgRNA efficiency in common bean. We compared the predicted efficiency scores of the designed sgRNAs with the *in planta* editing efficiencies observed in the transformed hairy roots. Moreover, we examined whether the predicted insertions and deletions (indels) generated by these computational programs matched the indels observed in the transformed hairy roots. This comparison allowed us to assess the accuracy and reliability of these *in silico* tools for predicting CRISPR/Cas9 editing outcomes in a plant system, specifically in common bean. Through this comprehensive approach, we not only identified preferred methods for the use of the CRISPR/Cas9 technology in common bean but also provided valuable insights for future research aimed at developing stable transgenic lines with improved nutritional quality for human and animal consumption.

## 2 Materials and methods

### 2.1 Construction of CRISPR/Cas9 vectors

#### 2.1.1 sgRNA design

The gene editing tool CRISPR/Cas9 was used to knockout the RFO biosynthetic genes, *Raffinose synthase 1*, *Raffinose synthase* 2 and *Stachyose synthase*, in common bean (de Koning et al., 2021; de Koning et al., 2023). The bioinformatics program CRISPOR (http://crispor.tefor.net/) was used to design sgRNAs targeting the 5-65% region of the coding sequence to avoid target sites near the 5’ or 3’ end of the protein coding region. This mitigates the possibility of an alternative start codon usage downstream of the initial ATG side thus increasing the probability of achieving a null mutation (Concordet and Haeussler, 2018). An *Arabidopsis thaliana* U6-26 promoter was used to drive the expression of the sgRNAs which requires a guanine at the base of transcription (Li et al., 2007; Li et al., 2018). Potential target sequences needed to be located directly next to the NGG protospacer adjacent motif (PAM) sequence required for the *Streptococcus pyogenes* Cas9 (SpCas9) enzyme (Jinek et al., 2012). Potential off-target sites were predicted by the bioinformatic programs CRISPOR and Cas-OFFinder (http://www.rgenome.net/cas-offinder/) which both used the *Phaseolus vulgaris* V2.1 as a reference genome (Bae et al., 2014b; Concordet and Haeussler, 2018). Off-target sequences with more than 4 mismatches including one in the seed region were dismissed (Modrzejewski et al., 2020). Furthermore, the impact of a DSB in an off-target region on the normal function of the common bean plant was investigated by analyzing the genomic sequence of the off-target site using the basic local alignment search tool (BLAST) against the annotated genome *P. vulgaris* v2.1 in the Phytozome database (Goodstein et al., 2012). All sgRNAs potentially targeting important off-target regions were discarded, as well as sgRNAs that contained a BbsI recognition site. Considering these requirements, sgRNAs were selected based on the highest predicted efficiency scores and lowest potential off-targets. An overview of the designed sgRNAs can be found in **Supplementary Table S1**.

#### 2.1.2 Entry vector construction

pEn-C1.1 vector (RRID : Addgene_61479) was used as entry vector which carries the sgRNA scaffold and RNA polymerase III U6 promoter to drive the expression of the sgRNA (Schiml et al., 2014). To incorporate a sgRNA into the BbsI site of the pEn-C1.1 entry vector a cut-ligation reaction was used. The final entry clones were transformed into competent *E. coli* DH5α cells using heat shock. To check if the sgRNA was incorporated in the final pEn-C1.1 vector correctly, diagnostic PCR was performed using primers listed in **Supplementary Table S2**.

#### 2.1.3 Destination vector construction

The destination vectors pMR356 and pMR394, derivatives of pMR290, were kindly provided by Dr. Mily Ron and Prof. Anne Britt of the Dept. of Plant Biology at the University of California, Davis (Bari et al., 2019). These vectors contain an *A. thaliana* codon-optimized Cas9 gene (*Atco-Cas9*), driven by either a parsley ubiquitin promoter (PcUbi(P)) in pMR356 or a 2x35S-Ω promoter (35S promoter featuring a duplicated enhancer and an omega translational enhancer from the tobacco mosaic virus) in pMR394. For this study, both vectors were further improved by the incorporation of an enhanced green fluorescent protein gene (*eGFP*), under the control of a 35S promoter and terminated by a nopaline synthase terminator (NOS ter) with polyadenylation signal (transcription unit originating from the pGFPGUSPlus vector; RRID: Addgene_64401), using the NEBuilder HiFi DNA Assembly master mix (New England Biolabs, Ipswich, Massachusetts, United States of America). The two new destination vectors were named respectively pMR356-GFP and pMR394-GFP and were transformed into competent *E. coli* DH5α cells using heat shock (**Supplementary Figures S1**, **S2**). Diagnostic PCR was used to check the correctness of the vectors using primers listed in **Supplementary Table S3**. For each sgRNA, a Gateway^™^ LR Clonase^™^ reaction (Thermo Fisher Scientific, Waltham, USA) was performed to incorporate them into the two destination vectors. The final expression clones were transformed into competent *E. coli* DH5α cells using heat shock. To confirm the correct recombination outcome, diagnostic PCR was performed with primers listed in **Supplementary Table S3**. Final expression clones were transformed into electrocompetent *R. rhizogenes* K599 cells using a Gene Pulser (Bio-Rad Laboratories) for electroporation. To confirm successful transformation, diagnostic PCR was performed with primers listed in **Supplementary Table S3**.

### 2.2 Plant material

*Phaseolus vulgaris* cv. CIAP7247F, cv. Pinto and cv. Rosecoco were grown in the greenhouse (Brussels, Belgium) with a 16h:8h day/night cycle to obtain fresh seeds. Harvested seeds were surface sterilized in 50 ml centrifuge tubes by submerging them in 30 ml of 70% (v/v) ethanol for 10 minutes at room temperature while being shaken at 100 rpm. Subsequently, the seeds were rinsed 5 times with sterile distilled water after which the seeds were submerged in 30 ml of a 20% (v/v) dilution of commercial bleach (final sodium hypochlorite concentration of 1%) complemented with 2 drops of polysorbate 20 and incubated at room temperature for 30 minutes while being shaken at 100 rpm. The seeds were rinsed 5 times with sterile distilled water after which they were soaked in sterile distilled water for 24 hours. The sterilized seeds were grown on Steri Vent high containers (Duchefa, CAT #S1686) containing germination media (1/2 strength Murashige and Skoog (MS) salts, 1 mg/l thiamine, 30 g/l sucrose, 6 g/l agar, pH 5.7) for 3 to 5 days in the dark at 24°C after which the plants were used for the transformation with *R. rhizogenes* K599. Alternatively, sterilized seeds were sown directly into soil-containing pots and grown at 24°C in a culture room or greenhouse with a 16h:8h day/night cycle until they were used for transformation with *R. rhizogenes* K599.

### 2.3 Plant transformation with *R. rhizogenes* K599

#### 2.3.1 Method 1: transformation by injection into the cotyledonary node

Five-day-old seedlings of *P. vulgaris* cv. CIAP7247F, grown in soil-containing pots in a culture room, as described in 1.2, were transformed with *R. rhizogenes* K599 using a method adapted from Estrada-Navarrete et al. (2006). Initially, *R. rhizogenes* K599 cultures with and without pGFPGUSPlus vector (RRID: Addgene_64401) were freshly grown in 40 ml liquid LB (supplemented with 50 μg/ml kanamycin for the culture containing pGFPGUSPlus) at 28°C and 200 rpm for 2 days until an OD_600_ of 1.2-1.7 was reached. Subsequently, the cultures were centrifuged at 3200 g for 10 min after which the pellets were resuspended in liquid MS medium (pH 5.5) and diluted to an OD_600_ of 0.5. The *β-glucuronidase* gene from the pGFPGUSPlus vector contains a castor bean catalase intron, ensuring expression exclusively in plant cells and preventing expression in bacteria. The cotyledonary nodes of 60 five-day-old seedlings were inoculated three times at different positions with 5 µl of the bacterial suspension containing pGFPGUSPlus, using a sterile syringe and needle. In addition, 10 five-day-old seedlings were transformed with *R. rhizogenes* K599 and 10 five-day-old seedlings were mock-infected with liquid MS medium as controls. The seedlings were then covered with transparent plastic containers and placed back into the culture room at 24°C and a 16h:8h day/night cycle.

#### 2.3.2 Method 2: transformation by inoculating an incision of the abaxial surface of the cotyledon

*P. vulgaris* cv. CIAP7247F seedlings were grown on a germination medium for 3 days in the dark and subsequently 4 days in a 16h:8h day/night cycle regime at 24°C, as described in 1.2, after which they were transformed with *R. rhizogenes* K599 using a method adapted from Keyes et al. (2009). The cotyledons were excised and a 1 mm deep diamond-shaped incision was made on the adaxial side of the cotyledon near the cotyledonary node, exposing but not slicing the midrib of the cotyledon. The cotyledons were placed on soaked sterile Whatman filter paper containing ¼ strength MS medium.

In advance, *R. rhizogenes* K599 cultures with and without vector pGFPGUSPlus (RRID: Addgene_64401) were freshly grown in 40 ml liquid LB (supplemented with 50 μg/ml kanamycin for the culture containing pGFPGUSPlus) for 2 days at 28°C and 200 rpm until an OD_600_ of 1.2-1.7 was reached. Subsequently, the cultures were centrifuged at 3200 g for 10 min after which the pellet was resuspended in bacterial inoculation media (¼ strength MS, 3.9 g/l MES, pH 5.5) and diluted to an OD_600_ of 0.5. Next, 200 µM acetosyringone was added and the bacterial cultures were incubated at 28°C and 200 rpm in the dark for 2 hours. Subsequently, 20 µl of the bacterial culture was applied to the diamond-shaped wound of each cotyledon. The cotyledons were incubated in the dark at 24°C. If required, ¼ MS was added to maintain the moisture of the Whatman filter paper. To avoid the decay of cotyledons, the Whatman filter paper should not be excessively wet. In total, 348 cotyledons were transformed with *R. rhizogenes* K599 (pGFPGUSPlus). For control, 30 cotyledons were transformed with *R. rhizogenes* K599, and 30 cotyledons were mock-infected with bacterial inoculation media.

#### 2.3.3 Method 3: transformation by inoculating a severed radicle still attached to the rest of the seedling

The protocol of Khandual and Reddy (2014) was optimized to induce transgenic hairy roots. The seed coats of 5-day-old seedlings of *P. vulgaris* cv. CIAP7247F, cv. Pinto and cv. Rosecoco, grown on germination media in a dark culture room, as described in 1.2, were removed after which the radicles were horizontally cut off at the base of the cotyledonary node while maintaining the two cotyledons attached. Additionally, small incisions were made at the cut site. In advance, *R. rhizogenes* K599 containing vectors pMR356-GFP or pMR394-GFP with relevant sgRNAs were grown on LB agar containing 50 μg/ml spectinomycin and 100 μg/ml streptomycin for 3 days at 28°C. Similarly, *R. rhizogenes* K599 was grown on LB agar without antibiotics. Freshly grown *R. rhizogenes* bacteria were scraped from the LB agar plate and applied to the cut site with a sterile spatula. The explants were then transferred to Steri Vent high containers containing germination media and incubated at 24°C with a 16h:8h day/night cycle for 5 days. A detailed video of the transformation procedure is available at Zenodo.org (https://doi.org/10.5281/zenodo.7943917). The explants were washed with sterile water supplemented with 50 mg/l kanamycin and 250 mg/l cefotaxime. The explants were then transferred to new Steri Vent high containers containing germination media supplemented with 250 mg/l cefotaxime, and 50 mg/l kanamycin and incubated at 24°C with a 16h:8h day/night cycle in a culture room. In total, 300 P*. vulgaris* cv. CIAP7247F explants, 141 P*. vulgaris* cv. Rosecoco explants and 79 P*. vulgaris* cv. Pinto explants were transformed with *R. rhizogenes* K599 containing vectors pMR356-GFP or pMR394-GFP with relevant sgRNAs. Additionally, 30 explants of each cultivar were transformed with *R. rhizogenes* K599 and mock-infected with sterile water as controls.

#### 2.3.4 Transgenic hairy root detection

Hairy roots were screened for the presence of green fluorescent proteins (GFP) under the Stereo Microscope Fluorescence Adapter system (NIGHTSEA, Lexington, USA) with the Royal Blue excitation head (440 – 460nm) in combination with the Green Bandpass Barrier Filter which transmits light from 500 to 560nm. GFP-positive hairy roots were indicative of the successful transformation of the *eGFP* reporter gene, which was linked to the CRISPR/Cas9 construct (pMR356-GFP or pMR394-GFP) or *β-glucuronidase* gene (pGFPGUSPlus), into the explants. Transgenic hairy roots longer than 3 cm were cut off at the base of the root, flash-frozen in liquid nitrogen and stored at -80°C. Additionally, hairy roots that emerged after the transformation with *R. rhizogenes* K599 (pGFPGUSPlus) were screened for the expression of β-glucuronidase (GUS) using the histochemical X-Gluc assay adapted from Jefferson et al., 1987. Hairy roots were placed in a centrifuge tube containing 5 ml ice-cold 90% acetone and incubated for 15 minutes on ice. The acetone was discarded, and the hairy roots were incubated for 10 min at room temperature in 5 ml sodium phosphate buffer (0.1 M NaH_2_PO_4_/Na_2_HPO_4_, pH 7). The sodium phosphate buffer was discarded and 5 ml of X-Gluc staining buffer (2 mM 5-bromo-4-chloro-3-indolyl-β-D-glucuronic acid, 10 mM EDTA, 1 mM K_3_Fe(CN)_6_, 1 mM K_4_Fe(CN)_6_, 0.1 M NaH_2_PO_4_/Na_2_HPO_4_ pH7) was added and the mixture was incubated overnight at 37°C. Finally, the X-Gluc staining buffer was discarded and the roots were placed in new centrifuge tubes containing 5 ml of 70% ethanol after which the stereo microscope Motic SMZ-161 (Motic, Kowloon City, Kowloon, Hong Kong) was used to visualize the blue-stained hairy roots.

### 2.4 Analysis of gene editing events

#### 2.4.1 DNA isolation

Individual transgenic hairy roots, as well as control roots, were crushed with a TissueLyser II (Qiagen, Hilden, German), after which genomic DNA was isolated using the NucleoSpin^™^ Plant II kit (Macherey-Nagel, Dueren, Germany, CAT #740770.50). The quality and quantity of the DNA samples were measured using a NanoDrop 1000 Spectrophotometer (Thermo Fisher Scientific).

#### 2.4.2 PCR

Primers were designed to amplify the target region of the sgRNAs, spanning an area of approximately 200 bp flanking the CRISPR/Cas9 cut site (**Supplementary Table S4**). PCR was performed using the GoTaq® G2 Green Master Mix (Promega). The PCR products were run on a 1.5% agarose gel and were visualized using the Gel Doc™ EZ Imaging System (Bio-Rad). Subsequently, the PCR products were cleaned using the Wizard^®^ SV Gel and PCR Clean-Up System (Promega, CAT #A9281) and outsourced for Sanger sequencing to Macrogen Europe (Amsterdam, Netherlands).

#### 2.4.3 sgRNA efficiency

The sgRNA efficiencies to induce insertions and deletions (INDEL score) and out-of-frame or knockout mutations (KO score) in the target region were determined both *in silico* and *in planta*. To determine the sgRNA efficiencies and mutational outcomes *in silico*, the bioinformatic programs CRISPOR, CRISPR RGEN and inDelphi were used (Park et al., 2015; Concordet and Haeussler, 2018; Shen et al., 2018). The CRISPOR and CRISPR RGEN programs used the *Phaseolus vulgaris* V2.1 as the reference genome and a 5’-NGG-3’ SpCas9 as PAM type. The sgRNA efficiencies are predicted by 4 different scores. The Doench/Fusi 2016 predicted efficiency score is based on data from mutational events resulting from CRISPR/Cas9-mediated cleavage of target sequences in human (MOLM13, NB4, TF1) and mice (BV2) cell lines transduced via lentiviral infection with sgRNAs transcribed by a U6 promotor (Doench et al., 2016). The CRISPRscan predicted efficiency score is based on data from mutational events resulting from CRISPR/Cas9-mediated cleavage of target sequences in zebrafish 1-cell stage embryos injected with *in vitro* transcribed Cas9 mRNA and *in vitro* transcribed sgRNAs using a T7 promoter (Moreno-Mateos et al., 2015). The logistic regression model to predict insertions and deletions (Lindel out-of-frame) score is based on data from mutational events (both deletions and insertions) resulting from CRISPR/Cas9-mediated cleavage of target sequences in a human cell line (HEK293T) transduced via lentiviral infection with sgRNAs. This model was trained to take into account microhomologies present near the target site (Chen et al., 2019). The Bae out-of-frame score is based on data from mutational events (only deletions) resulting from TALEN and CRISPR/Cas9-mediated cleavage of target sequences in human cell lines (HEK293T and K562) after transfection of a Cas9-encoding plasmid and *in vitro* transcribed sgRNAs using a T7 promoter. Microhomologies in the sequences flanking the target site were used to predict the Bae out-of-frame score (Bae et al., 2014a; Park et al., 2015). The inDelphi algorithm can predict CRISPR/Cas9 outcomes and efficiencies based on the flanking sequences of the cut site of the designed sgRNA. The algorithm was initially trained on mutational events (insertions as well as deletions) resulting from CRISPR/Cas9 cleavage of human (U2OS) and mouse (mESCs) cell lines generated by a Tol2 transposon-based SpCas9 expression plasmid as well as data from human cell lines HEK293, K562 and HCT116. Furthermore, the machine learning model uses a module that simulates the MMEJ repair mechanism and can predict the microhomology strength of the flanking sequences (Shen et al., 2018). All efficiency scores of the previously mentioned computational programs are rated from 0 to 100, with a higher score being better. Furthermore, the Lindel and Bae repair models, as well as the inDelphi algorithm were used to predict the CRISPR/Cas9 mutational outcomes.

To determine the *in planta* INDEL and KO scores, the bioinformatics program Tracking of Indels by Decomposition (TIDE) was used (Brinkman et al., 2014). The TIDE software predicts which specific indels were present in a Sanger sequence read of an edited sample in comparison with a wild-type sequence. The maximum size of the insertions and deletions was set to 30 bp. The TIDE software displays the predicted indels as a percentage, which corresponds to the percentage of DNA sequences that contain that specific indel. Only indels with a *p*-value lower than 0.01 were used in the calculation of the INDEL and KO scores of each hairy root. Furthermore, only hairy root samples for which the proposed indel distribution fitted the Sanger sequence data with an R^2^ value higher than 0.7 were used. At least fifteen hairy roots were analysed for each sgRNA in combination with the pMR356-GFP and pMR394-GFP vectors. The INDEL and KO scores were first calculated for each hairy root, after which the average INDEL and KO score was calculated using Microsoft Excel (v16). The INDEL score for a hairy root is the sum of the percentages of predicted indels for that root. The KO score for a hairy root is the sum of the percentages of indels that cause a frameshift or indels which are equal to or larger than 21 base pairs, which in both cases are likely to generate a complete loss-of-function mutation.

## 3 Results

### 3.1 Screening for an effective protocol to generate transgenic hairy roots

Three different methods were tested for hairy root transformation of *P. vulgaris*. These methods were evaluated based on the percentage of transformed explants forming GFP-positive hairy roots (i.e. roots containing the T-DNAs of both the Ri plasmid and binary vector), the abundance of hairy roots emerging on one explant and the speed of hairy root appearance. An overview of the results can be found in **Table 1**.

**Table 1:** Overview of the efficiency of three different transformation methods to induce GFP-positive hairy roots in *P. vulgaris* using *R. rhizogenes* K599 containing pGFPGUSPlus or pMR356-GFP with a CRISPR construct.

Transformation method	*P. vulgaris* cultivar	Explants having GFP-positive hairy roots/total explants	% of explants that have GFP-positive hairy roots	Amount of GFP-positive hairy roots per explant [low (<5), medium (5-10), high (>10)]	Emergence time of GFP-positive hairy roots (days after infection)
Method 1	CIAP7247F	21/60	35.0	low-medium	35
Method 2	CIAP7247F	53/348	15.2	low	15
Method 3	CIAP7247F	144/300	48.0	medium-high	15
Method 3	Pinto	33/79	41.8	medium-high	20
Method 3	Rosecoco	58/141	41.1	medium-high	20

The transformation was based on the injection of the cotyledonary node for method 1, inoculating an incision of the adaxial surface of the cotyledon for method 2 and inoculating a severed radicle still attached to the rest of the seedling for method 3. Only hairy roots that were GFP positive or stained blue after a histochemical X-Gluc assay were counted.

#### 3.1.1 Method 1: transformation by injection into the cotyledonary node

**Figure 1:**
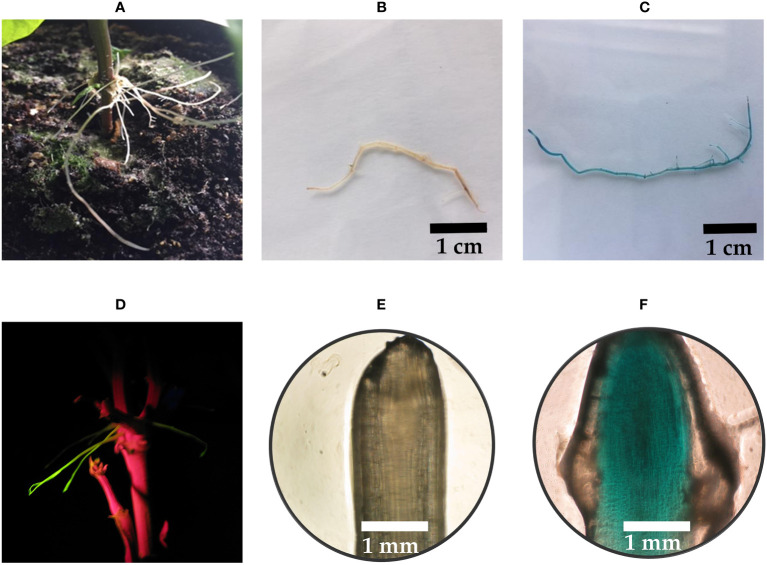
*R. rhizogenes* transformation by injection into the cotyledonary node of *P. vulgaris*. **(A)** Hairy roots at the cotyledonary node, 45 days after injection. **(B)** Negative control root after a histochemical X-Gluc assay. **(C)** Blue-colored transgenic hairy root after a histochemical X-Gluc assay. **(D)** Hairy roots showing fluorescence emission of GFP further along the stem. **(E)** Root tip of a negative control after a histochemical X-Gluc assay under 50x magnification. **(F)** Root tip of a GFP-positive hairy root after a histochemical X-Gluc assay under 50x magnification.

To investigate the efficiency of hairy root induction by injecting the cotyledonary node of five-day-old seedlings, 60 plants were transformed with *R. rhizogenes* K599 (pGFPGUSPlus). After 35 days, 21 out of the 60 plants had GFP-positive hairy roots, yielding a transformation efficiency of 35% (**Table 1**). The GFP-positive hairy roots also showed blue staining after undergoing a histochemical assay with X-Gluc (**Figures 1B, C, E, F**). Each plant induced a low (<5) to medium (5 to 10) number of hairy roots (**Figure 1A**). Many of these hairy roots appeared at the cotyledonary node. However, GFP-positive hairy roots also appeared further along the stem (**Figure 1D**). Besides GFP-positive hairy roots, a high number of GFP-negative roots also appeared at the side. The emergence time of hairy roots was around 35 days after injection.

#### 3.1.2 Method 2: transformation by inoculating an incision of the adaxial surface of the cotyledon

**Figure 2:**
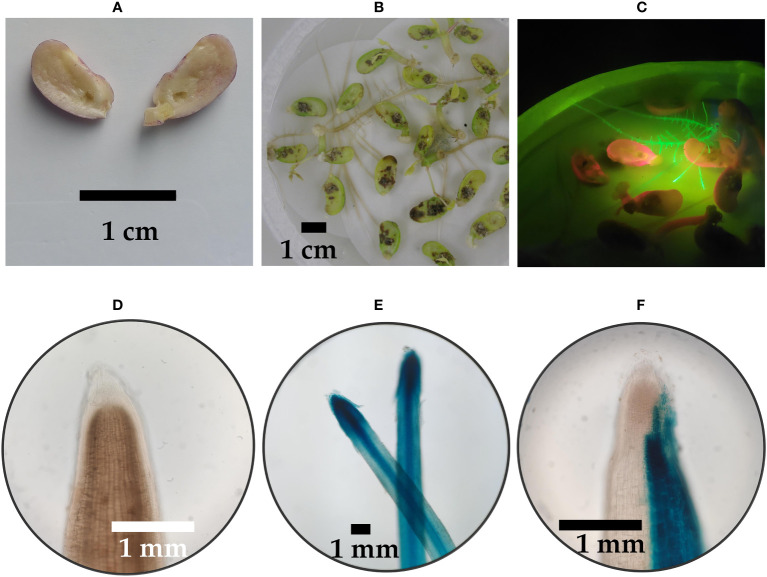
Transformation of *P. vulgaris* by inoculation of an incision on the adaxial surface of a cotyledon. **(A)** Cotyledons with a 1 mm deep diamond-shaped incision **(B)** Hairy roots emerging, 18 days after infection **(C)** Transgenic hairy roots showing fluorescence emission of GFP **(D)** Root tip of a negative control after a histochemical X-Gluc assay under 50x magnification. **(E)** Two root tips of transgenic hairy roots after a histochemical X-Gluc assay under 20x magnification **(F)** Root tip of a partially transgenic hairy root after a histochemical X-Gluc assay under 50x magnification.

Explants transformed by inoculating an incision of the adaxial surface of the cotyledon yielded a transformation efficiency of 15.2% (**Figure 2A**; **Table 1**). Of the 348 transformed explants, 53 contained hairy roots that showed fluorescence emission of GFP while also GFP-negative roots were observed (**Figure 2C**). The GFP-positive hairy roots also stained blue after a histochemical X-Gluc assay (**Figures 2D, E**). However, not all fluorescent hairy roots stained completely blue as seen in **Figure 2F**. Around 15 days after inoculation, the first hairy roots emerged. Each explant induced a low (<5) number of hairy roots (**Figure 2B**).

#### 3.1.3 Method 3: transformation by inoculating a severed radicle still attached to the seedling

**Figure 3:**
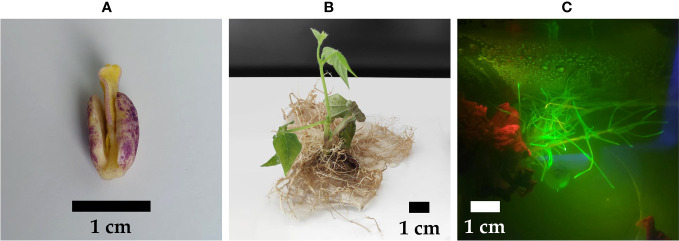
Transformation of *P. vulgaris* by inoculation of a severed radicle still attached to the rest of the seedling. **(A)** Explant with a severed radicle **(B)** Explant with hairy roots, 35 days after infection **(C)** Transgenic hairy roots showing fluorescence emission of GFP.

In our initial exploration, we conducted experiments using the pGFPGUSPlus vector for Method 3, which exhibited superior performance when compared to the other two methods. Consequently, we proceeded to employ the pMR356-GFP and pMR394-GFP vectors, given their capability to yield hairy roots suitable for subsequent experimental phases. A total of 520 P*. vulgaris* explants were transformed by inoculating a severed radicle with *R. rhizogenes* K599 containing either pMR356-GFP or pMR394-GFP with CRISPR constructs (**Figure 3A**). Among them, 144 of the 300 explants from cv. CIAP7247F had fluorescent hairy roots emerging from the cut side, which represented a transformation efficiency of 48% (**Table 1**; **Figure 3C**). The transformation efficiencies for cv. Pinto and cv. Rosecoco were 42% (33/79) and 41% (58/141), respectively. The average emergence time for hairy roots was around 15 days after inoculation for cv. CIAP7247F and around 20 days for cv. Pinto and cv. Rosecoco. Each explant induced a high (>10) number of hairy roots on average (**Figure 3B**). Along with GFP-positive hairy roots, a few GFP-negative roots also emerged from the cut side.

### 3.2 Construction of CRISPR/Cas9 vectors

**Figure 4:**
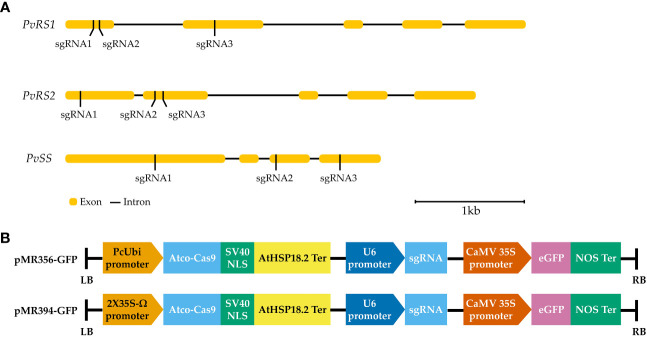
**(A)** Position of the sgRNAs targeting the raffinose synthase (*PvRS1* and *PvRS2*) and stachyose synthase (*PvSS*) genes in *P. vulgaris*. **(B)** Schematic representation of the T-DNA of the pMR356-GFP and pMR394-GFP vectors. The *A. thaliana* codon-optimized Cas9 gene (*Atco-Cas9*) was driven by a Parsley Ubiquitin promoter (PcUbi) in pMR356-GFP whereas *Atco-Cas9* was driven by a 2X35S-Ω promoter in pMR394-GFP. In both vectors, the *Atco-Cas9* gene was fused to a nuclear localization signal derived from simian virus 40 (SV40 NLS) and transcription was terminated by a *A. thaliana* heat shock protein 18.2 terminator (AtHSP18.2 ter). The transcription of the sgRNA was driven by a U6 promoter. The *enhanced green fluorescent protein* (*eGFP*) gene was placed under the regulation of a Cauliflower Mosaic Virus 35S promoter (CaMV 35S) and nopaline synthase terminator (NOS ter).

The raffinose family oligosaccharides biosynthetic genes of common bean were chosen as targets to test the efficiency of CRISPR/Cas9 in *P. vulgaris* (de Koning et al., 2021). Three different sgRNAs were designed to target the exon region of each gene (**Figure 4A**). The designed sgRNAs were selected based on the highest predicted efficiency scores and lowest potential off-targets. For *raffinose synthase 1* (*PvRS1*), exon 1 was targeted by two sgRNAs (sgRNA1 and sgRNA2) and exon 2 was targeted by one sgRNA (sgRNA3). For *raffinose synthase 2* (*PvRS1*), exon 1 was targeted by one sgRNA (sgRNA1) and exon 2 was targeted by two sgRNAs (sgRNA2 and sgRNA3). The *stachyose synthase* (*PvSS*) gene was targeted in exon 1, exon 3 and exon 4, each by one sgRNA (sgRNA1, sgRNA2 and sgRNA3, respectively). Initially, each sgRNA was first ligated into an entry vector followed by a Gateway^™^ LR Clonase^™^ reaction to obtain two expression clones, pMR356-GFP and pMR394-GFP both containing the same sgRNA (**Figure 4B**). The destination vectors differed in the promoter driving the *A. thaliana* codon-optimized Cas9 gene expression, namely PcUbi (pMR356-GFP) and 2X35S-Ω (pMR394-GFP). The pMR356-GFP and pMR394-GFP vectors were initially improved by the incorporation of an enhanced green fluorescent protein gene (*eGFP*) for visual distinction of transgenic hairy roots. In total, 18 different CRISPR/Cas9 constructs were developed to determine the gene editing efficiency of the designed sgRNAs in *P. vulgaris*.

### 3.3 *In silico* predicted sgRNA efficiencies

For each sgRNA, CRISPOR, CRISPR RGEN and inDelphi generated *in silico* predicted sgRNA efficiency scores ranging from 0 to 100, with higher scores indicating higher efficiency. The predicted on-target activity of the sgRNAs differed substantially between sgRNAs (**Table 2**). The efficiency scores as predicted by the Doench/Fusi 2016 model ranged from 36 to 69 whereas the CRISPRscan scores ranged from 32 to 78. The out-of-frame scores predicted by the Bae model, which only considers potential deletions, ranged from 51.0 to 79.6, while the Lindel out-of-frame scores, which includes deletions as well as insertions, were higher and ranged from 69 to 90. The inDelphi frameshift frequency also considers insertions as well as deletions in the prediction of mutation outcomes and ranged from 47.1 to 83.0. Furthermore, for the same sgRNA significant differences were observed between the predicted efficiency scores for the different test models. For instance, sgRNA1 targeting *PvRS1* had the highest Lindel score of 90 but only obtained a CRISPRscan score of 43 and an inDelphi frameshift frequency of 55.7.

**Table 2:** *In silico* results of the predicted sgRNAs efficiency scores generated from CRISPOR, CRISPR RGEN and inDelphi models.

Target	sgRNA	CRISPOR			CRISPR RGEN	inDelphi
		Doench/Fusi 2016 score	CRISPRscan score	Lindel out-of-frame score	Bae out-of-frame score	Frameshift frequency
*PvRS1*	1	69	43	90	79.6	55.7
	2	63	49	73	60.0	47.1
	3	55	50	69	57.0	67.1
*PvRS2*	1	67	53	77	73.7	81.7
	2	36	78	77	64.0	69.0
	3	68	51	76	61.3	61.3
*PvSS*	1	60	51	76	63.2	66.4
	2	42	32	78	68.3	78.8
	3	61	44	83	51.0	83.0

Scores are rated from 0 to 100. Higher scores indicate higher efficiency. Pink shading: *PvRS1*; green shading *PvRS2*; grey shading: *PvSS*.

### 3.4 Editing efficiencies of the sgRNAs under influence of varying promoters

**Figure 5:**
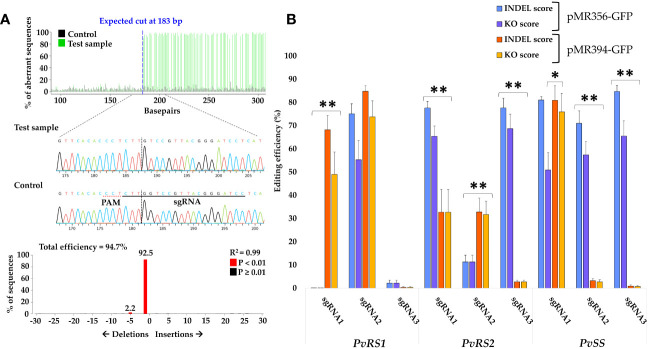
Detection of mutations and summary of editing efficiency of 9 different sgRNAs within pMR356-GFP and pMR394-GFP targeting the RFO biosynthetic genes in *P. vulgaris*. **(A)** Overview of TIDE results, including observed mutation types, of one hairy root edited by sgRNA3 (pMR356-GFP) targeting *Raffinose synthase 2* (*PvRS2*). **(B)** Summary of the mean sgRNA efficiencies in inducing insertions and deletions (INDEL score) and knockout mutations (KO score) in *Raffinose synthase 1* (*PvRS1*), *PvRS2* and *Stachyose synthase* (*PvSS*). The mean INDEL and KO scores for each sgRNA of pMR356-GFP and pMR394-GFP are shown. Error bars represent the standard error of the mean (n ≥ 15). A single asterisk (*) indicates that only the KO scores were significantly different (p < 0.05). Double asterisk (**) indicates that both the INDEL scores as well as the KO scores were significantly different (p < 0.01) based on a two-sample t-test assuming unequal variances. All gene editing analyses were performed on hairy roots derived from Method 3.

All gene editing analyses described herein were performed on the hairy roots derived from Method 3, as described above. To assess the efficiency of the designed sgRNAs and the influence of different promoters driving Cas9, we analyzed high-quality Sanger sequences of the target region of transgenic hairy roots for the presence of indels using TIDE. The majority of the designed sgRNAs caused mutations in the target sequence. This indicates that the Cas9 nuclease successfully cleaved DNA at the target site, triggering DNA repair and causing potential sequence alterations (**Figure 5A**). For each sgRNA, at least 15 hairy roots were analyzed for the presence of indels after which the mean INDEL score and KO score were calculated (**Figure 5B**; **Supplementary Table S5**). Our results indicate that not all designed sgRNAs had high gene editing efficiencies (mean INDEL and KO scores) and that the promoter driving Cas9 expression also influenced the cleavage efficiency. For *PvRS1*, sgRNA2 had the highest mean INDEL and KO scores, with respective values of 75.1% (SE = ± 4.3) and 55.3% (SE = ± 8.3) when paired with Cas9 under the PcUbi promoter (pMR356-GFP) and respective values of 84.8% ( ± 2.4) and 73.8% ( ± 6.9) when paired with Cas9 under the 2X35S-Ω promoter (pMR394-GFP). For *PvRS2*, sgRNA1 and sgRNA3 resulted in high gene editing efficiency scores with Cas9 under the PcUbi promoter (pMR356-GFP), with mean INDEL scores of 77.6% and KO scores ranging from 65.4 to 68.7%. The highest editing efficiency for *PvSS* was obtained by sgRNA3 with Cas9 under the PcUbi promoter, with a mean INDEL score of 84.7% (SE = ± 2.6) and a mean KO score of 65.5% (SE = ± 6.6). For four sgRNAs, significantly higher efficiency scores were obtained when Cas9 was driven by the PcUbi promoter, while for only two sgRNAs, Cas9 under the 2X35S-Ω promoter performed significantly better. For the remaining three sgRNAs, no significant difference was found between the two Cas9 promoters. The sgRNA3 targeting *PvRS1* had very low INDEL and KO scores when paired with Cas9 under the pcUbi or the 2X35S-Ω promoter. This observation can be attributed to the presence of a single nucleotide polymorphism (SNP) in the seed sequence right next to the PAM site which caused a mismatch with the sgRNA. This SNP in the target gene is specific for *P. vulgaris* cv. CIAP7247F as confirmed by sequencing of the target region and was not present in the reference genome (*P. vulgaris* cv. G19833).

### 3.5 Analysis of mutation diversity and frequency

**Figure 6:**
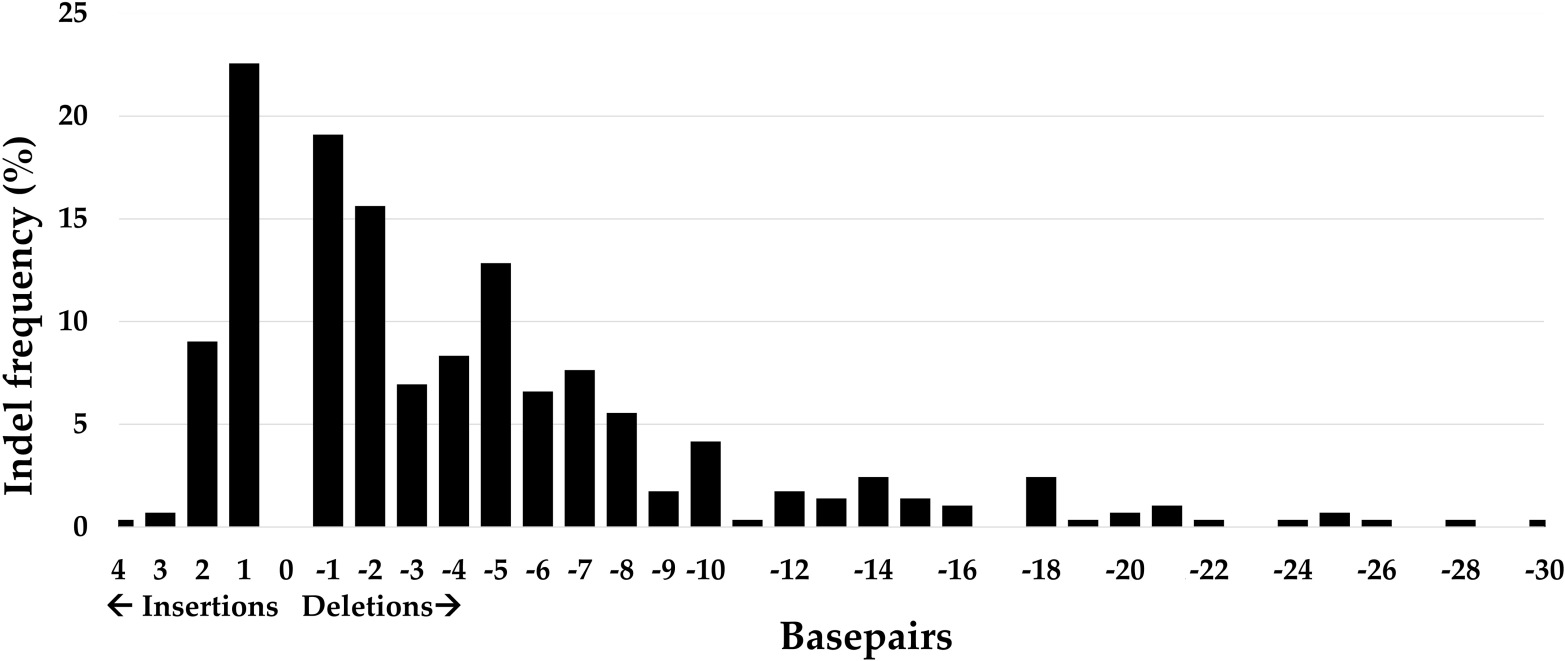
Summary of all mutation types and their frequency of occurrence in 288 hairy roots of *P. vulgaris* derived from Method 3 and induced by 18 different CRISPR/Cas9 constructs. One transgenic hairy root could contain different indels.

Within one hairy root, multiple dominant indels with an occurrence frequency higher than 20% could be seen. The number of dominant indels per hairy root varied for each sgRNA and ranged from one to four indicating that each sgRNA could induce different indels in one hairy root. For example, in the analyzed hairy roots edited by sgRNA1 targeting *PvRS2*, only one or two dominant indels were present per hairy root. Indels that occurred more than 20% in the hairy root sample pool for each sgRNA are listed in **Table 3**. In general, three times more deletions than insertions occurred within the 288 analyzed hairy roots (**Figure 6**). The most observed indels were one base pair (bp) deletions and insertions, with frequencies of 22.6 and 19.1%, respectively. The largest deletion observed was 30 bp long while the largest insertion observed was 4 bp long.

**Table 3:** Overview of the most occurring indels produced by sgRNAs targeting the RFO biosynthetic genes of common bean *in planta*, as well as the top 5, *in silico* predicted indels.

		pMR356-GFP	pMR394-GFP	CRISPOR	CRISPR RGEN	inDelphi	
Target	gRNA	Indels (bp) with >= 20% occurrence	Indels (bp) with >= 20% occurrence	Lindel repair model (top 5)	Bae repair model (top 5)	Predicted indels (top 5)	Similar outcomes *in silico* vs *in planta*
*PvRS1*	1	/	**-1**, -4	+1, **-1**, -8, +3, +1	-8, -10, -11, -7, -18	-3, **-1**, +1, -14, -9	**-1**
	2	-5, -6, **-2**	-10, -5, -7, **-4**	+1, -12, **-4**, **-2**, -2	-12, -15, -29, -32, -20	-12, **-2**, **-4**, +1, -9	**-2, -4**
	3	**+1**	-	**+1**, -3, -3, -7, +3	-21, -23, -17, -22, -27	-3, -7, **+1**, -10, -3	**+1**
*PvRS2*	1	**+1**, -8, -13, -6	+2	**+1**, -21, -2, -5, +3	-3, -4, -17, -21, -20	**+1**, -22, -17, -1, -1	**+1**
	2	-1, **-2**	-1, **-2**, **-5**	**-5**, -4, -16, **-2**, -3	-11, -12, -16, -11, -15	-3, +1, -4, -11, -12	**-2, -5**
	3	**-1**, -5, -7, **-8**, -6, **-2**, **+1**	**+1**	**+1, -1**, **-1**, **-8**, -9	**-2**, -9, -10, -15, -20	**+1**, **-6**, -9, -10, **-8**	**+1, -1, -2, -6, -8**
*PvSS*	1	**-3**, -14, -4, -2	-2, -4, **+1**	**+1**, **-3**, -11, -1, -1	-8, -11, -17, -18, -19	**-3**, **+1**, -11, -8, -10	**+1, -3**
	2	**+1**, **-1**, **-18**	**+1**, +2	-2, -5, **-1**, -10, **-18**	-11, **-18**, -10, -5, -19	-5, -11, **+1**, -10, -2	**+1, -1, -18**
	3	**+1**, +2, **-3**, -7, **-4**, **-1**	-	**-4**, **+1**, +3, **-1**, **-1**	-19, -27, -30, -30, -24	**-4**, **+1**, **-3**, **+1, -1**	**+1, -1, -3, -4**

Results are ordered from high to low occurrence. Indels that are depicted in green are indels seen both *in planta* and *in silico*. A mutation type can be predicted multiple times as long as the outcome is different, for example, a 1 bp insertion of different bases. The *in planta* analyses were performed on hairy roots derived from Method 3.

The computational models used to predict indels showed inconsistencies when compared to the observations made *in planta.* Specifically, the predictions of indels made by the Bae repair model were inconsistent with results observed in hairy roots, as indicated in **Table 3**. Out of the nine sgRNAs studied, only two produced indels that matched the predictions made by the Bae repair model. Only for sgRNA3 targeting *PvRS2* and sgRNA3 targeting *PvRS3* the Bae repair model predicted respectively a 2 bp deletion and an 18 bp deletion correctly. In contrast, the inDelphi algorithm and Lindel repair model performed more effectively, accurately predicting the most occurring *in planta* observed indels for the majority of sgRNAs. For instance, sgRNA3 targeting *PvSS* resulted in 6 different mutations that occurred in more than 20% of the analyzed hairy roots of which 4 mutations were correctly predicted by the inDelphi algorithm and 3 of them by the Lindel repair model. In contrast, the Bae repair model did not predict any of the observed indels in this case.

The CRISPR/Cas9 on-target activity led to a diverse array of indels at the designated target loci. To examine the potential involvement of MMEJ in the repair process, the microhomology strength (defined by the length of local microhomologies, their GC content, and position near the cleavage site) was assessed using the inDelphi algorithm. Out of the nine sgRNAs analyzed, six target sites exhibited an average microhomology strength. Notably, the target site of sgRNA2 targeting *PvRS1* and sgRNA2 targeting *PvRS2* demonstrated a high microhomology strength (**Table 4**). In contrast, the target site of sgRNA1 targeting *PvRS2* exhibited a low microhomology strength.

**Table 4:** Summary of the microhomology strength of the target sites of sgRNAs targeting the RFO biosynthetic genes of common bean and predicted and *in vivo* observed single nucleotide insertions at those loci.

		inDelphi		*In vivo*		
Target	sgRNA	Microhomology strength	Predicted 1 bp insertions	Hairy roots with insertions	Observed 1 bp insertions	Nucleotides flanking the cut site (| = cut site)
*PvRS1*	1	Average (0.5)	T (100%)	/	/	^5’^ TGT|GGC^3’^
	2	High (1.25)	T (53.3%), C (25.6%), A (21.1%)	2	C (48%), A (48%), T (4%)	^5’^ CTC TCT^3’^
	3	Averge (0.58)	C (100%)	3	A (50%), G (36.7%), T (13.3%)	^5’^ ATC|GTC^3’^
*PvRS2*	1	Low (0.23)	A (100%)	10	A (75.6%), C(18.9%), T (4.4%), G (1.1%)	^5’^ AAA|GGG^3’^
	2	High (1.09)	A (100%)	1	A (100%)	^5’^ GGA|GGG^3’^
	3	Average (0.41)	T (100%)	3	T (67.4%), C (27.8%), A (3.9), G (0.9%)	^5’^ CTT|GGT^3’^
*PvSS*	1	Average (0.48)	T (91.5%), A (8.5%)	3	T (68.3%), G (26.7%), A (3.3%), C (1.7%)	^5’^ GGT|AGT^3’^
	2	Average (0.49)	A (100%)	16	A (90.3%), G (6.6%), C (3.1%)	^5’^ GAA|CAC^3’^
	3	Average (0.54)	A (55%), T (29%), G (16%)	8	C (29.6%), T (28.8%), A (25.1%), G (15.3%)	^5’^ TGA|TTG^3’^

The microhomology strength was predicted based on the 100 bp sequences flanking both sides of the cut site. Observed one bp insertions that corresponded with the nucleotide at the -4 position upstream of the PAM were depicted in green. The *in vivo* analyses were performed on hairy roots derived from Method 3. Pink shading: *PvRS1*; green shading *PvRS2*; grey shading: *PvSS*.

Where MMEJ only leads to deletions, NHEJ can also result in insertions. Interestingly, the inDelphi algorithm accurately predicted the occurrence and nucleotide type of one base pair insertions in most instances. For five out of the nine sgRNAs, the *in vivo* outcomes aligned with the algorithm’s predictions. In these cases, the observed inserted nucleotide corresponded with the nucleotide at the -4 position upstream from the PAM site (**Table 4**). In two cases (sgRNA2 targeting *PvRS1* and sgRNA3 targeting *PvSS*), the predicted outcomes were partially correct. Only for sgRNA3 targeting *PvRS1*, the observed insertions did not correspond with the algorithm’s predictions.

## 4 Discussion

### 4.1 Optimizing hairy root transformation for *P. vulgaris* cv. CIAP7247F

Although several hairy root transformation protocols have been developed for common bean plants, we observed that the issue of genotype specificity continues to pose a challenge (Estrada-Navarrete et al., 2006; Khandual and Reddy, 2014; Voß et al., 2022). To find a preferred transformation protocol for generating transgenic hairy roots for *P. vulgaris* cv. CIAP7247F, we evaluated three distinct transformation protocols using *R. rhizogenes* K599. This strain was found to be very effective in inducing hairy roots not just in common bean plants, but also across a broad range of leguminous species (Estrada-Navarrete et al., 2006; Keyes et al., 2009; Khandual and Reddy, 2014; Xiang et al., 2016; Voß et al., 2022).

Method 1, adapted from Estrada-Navarrete et al. (2006), involved the injection of *R. rhizogenes* into the cotyledonary node of intact seedlings. This method resulted in a transformation efficiency of 35% and produced a low to medium number of GFP-positive hairy roots that emerged from the cotyledonary node and along the stem. This method had several limitations such as a lengthy waiting time for hairy root emergence of 35 days after infection, the emergence of a high number of GFP-negative roots at the cotyledonary node and along the stem, and the use of non-sterile growth conditions which could impact downstream processes. The transformation efficiencies reported by Estrada-Navarrete et al. (2006) using *R. rhizogenes* K599 ranged from 70% to 90%, which is higher than the 35% we observed for cultivar CIAP7247F. However, in the calculation for transformation efficiency, we only included explants that produced GFP-positive hairy roots that stained blue after a histochemical X-Gluc assay and did not include explants that produced only GFP-negative roots. In total 57% of our explants generated hairy roots of which 22% did not contain any GFP-positive hairy root. Explants containing GFP-positive hairy roots also showed numerous GFP-negative roots, which was also observed by Khandual and Reddy (2014). Their study reported that between 40% and 80% of the emerging roots did not contain T-DNA of the binary vector using the same method.

Method 2, adapted from Keyes et al. (2009), involved the inoculation of an incision created on the adaxial surface of the cotyledon. Although this approach was initially developed for soybean plants, its potential applicability to *P. vulgaris* was also considered. For common bean, this method resulted in relatively fast hairy root growth, with roots appearing just 15 days after inoculation. Additionally, the resulting hairy roots were developed in sterile conditions which could be beneficial for downstream processes. Nonetheless, only 15.2% of explants produced GFP-positive hairy roots, and the number of hairy roots per explant remained limited. A high incidence of cotyledon rot was also observed, which may pose a constraint for this technique. It is important to note that not all emerging hairy roots were GFP-positive, an observation also observed by Keyes et al. (2009), who reported that merely 55% of the developing roots contained T-DNA of the binary vector. Additionally, we noticed multiple instances where only one side of the root was stained following a histochemical X-Gluc assay (**Figure 2F**). This phenomenon might be attributed to either the early loss of transgenes (T-DNA not stably integrated in the genome) or loss of transgene expression (due to silencing) during the growth of a hairy root or the result of the inaccurate assumption that hairy roots are always derived from a single cell, rendering them chimeric, as observed in previous studies (Limpens et al., 2004; Garagounis et al., 2020). This observation is significant because it could impact the interpretation of gene editing events in hairy roots.

Method 3 was adopted from Khandual and Reddy (2014) and involved inoculating a severed radicle still attached to the rest of the seedling. For this method, the pMR356-GFP and pMR394-GFP vectors were used instead of the pGFPGUSPlus, which possess identical eGFP transcription units encompassing the same 35S promoter, *eGFP*, and NOS ter with polyadenylation signal. As a result, we anticipated a comparable level of *eGFP* expression for these three vectors. Additionally, since all experiments utilized the *R. rhizogenes* K599 strain for hairy root induction, uniformity in the Ri plasmid and chromosomal context is ensured. As a result, we expect consistent induction of hairy roots across all methods. The use of this method resulted in the highest transformation efficiency, with 41-48% of the explants carrying GFP-positive hairy roots. Hairy roots emerged relatively quickly (15-20 days after infection) and were obtained under sterile conditions. Furthermore, a high number of GFP-positive hairy roots were produced per explant. This technique proved effective in other *P. vulgaris* cultivars, such as Pinto and Rosecoco, demonstrating its capacity to induce hairy root growth in a broader range of common bean cultivars. One limitation of this method was the emergence of GFP-negative roots alongside the desired GFP-positive roots. This finding contrasts with the report by Khandual and Reddy (2014), which mentioned that all emerging hairy roots in their study contained T-DNA of the binary vector. However, the occurrence of GFP-negative roots was observed in all other tested methods and could potentially be the formation of normal roots originating from meristematic cells or callus cells at the cotyledonary node or the generation of hairy roots without incorporation of the T-DNA of the binary vector.

After evaluating the three different methods for hairy root transformation, Method 3 emerged as the most promising protocol for generating transgenic hairy roots in common bean. While our study does not involve a direct comparison of Method 3 with the other two methods, mainly due to variations in the transgenic constructs used, we are confident that these differences are minor. Consequently, we conclude that Method 3 demonstrated the highest transformation efficiency, rapid emergence of hairy roots, and the development of a significant number of GFP-positive hairy roots per explant. One drawback of all tested methods was the co-occurrence of GFP-negative alongside GFP-positive hairy roots, underscoring the need for additional screening steps to accurately identify hairy roots that contain the T-DNA of the binary vector. To address this, the incorporation of enhanced green fluorescent protein (eGFP) in our CRISPR/Cas9 vectors was necessary, allowing for a visual distinction of fluorescent hairy roots, which were subsequently used in further analysis.

### 4.2 Evaluating the sgRNA efficiency and promoter impact on CRISPR/Cas9 gene editing

The *in planta* effectiveness of *in silico*-designed sgRNAs that target genes involved in the RFO metabolic pathway of common bean were evaluated using the hairy root transformation system. Common bean has two raffinose synthase genes (*PvRS1* and *PvRS2*) and one stachyose synthase gene (*PvSS*) encoding RFO biosynthetic enzymes (de Koning et al., 2021). Targeting these genes with CRISPR/Cas9 to generate knockout lines with reduced quantities of RFOs in the seed could improve the nutritional quality of these beans and alleviate consumption-related discomforts (Coon et al., 1990; Tomlin et al., 1991; Valentine et al., 2017; de Koning et al., 2021). There are important factors to consider before embarking on the more time-consuming stable transformation experiments. These factors include the fact that the effectiveness of the CRISPR/Cas9 system is influenced by the editing efficiency and specificity of the sgRNA as well as the promoter driving Cas9 expression (Ma et al., 2015; Liang et al., 2016; Ma et al., 2016; Li et al., 2022). By comparing the efficiency of sgRNAs and the promoters driving Cas9 using the hairy root transformation system, we aimed to identify the most effective combination that can be used in future experiments. Both ICE and TIDE software can be used to analyze Sanger data for potential mutations. We observed a strong correlation between the results of both programs (data not shown), consistent with findings from other studies (Conant et al., 2022). However, one major advantage of TIDE over ICE is that it provides *p*-values of all predicted indels and allows for specific parameter adjustments. For each sgRNA, at least 15 hairy roots were analyzed for the presence of indels, after which the mean INDEL score and KO score were calculated. This provides a general overview of the efficiency of sgRNAs and promoters within different hairy roots as CRISPR/Cas9 can potentially function differently within distinct plantlets. Furthermore, the resulting outcome of a DSB is also dependent on the repair mechanism, which relies partially on the specific target sequence. Not all designed sgRNAs showed good *in planta* efficiency, highlighting the importance of initially testing the sgRNA efficiency with our hairy root system. Nonetheless, we identified at least one or two highly efficient sgRNAs for all target genes. A noteworthy discovery in this study was the observation of low INDEL and KO scores for sgRNA3 targeting the *PvRS1* gene, which was attributed to an SNP in the seed sequence immediately adjacent to the PAM site. This SNP led to a mismatch with the sgRNA sequence, resulting in a close to zero gene editing efficiency. This finding underscores the crucial need to screen for genetic variations within the targeted genomic regions of the chosen cultivars during sgRNA design, as SNPs or other sequence variations can have a substantial impact on gene editing outcomes (Modrzejewski et al., 2020). As shown here, only one mismatch in the seed sequence is enough to greatly reduce Cas9 cleavage, given the essential role the seed sequence plays in facilitating Cas9’s target sequence recognition (Modrzejewski et al., 2020). Furthermore, a difference in editing efficiency could be seen when Cas9 expression was driven by the PcUbi or the 2X35S-Ω promoter. In most cases, the PcUbi promoter resulted in higher INDEL and KO scores making it the preferred promoter to be used in common bean for further stable transformation experiments. On only two occasions did the 2X35S-Ω promoter result in higher scores. However, for three sgRNAs (sgRNA3 targeting *PvRS2*; sgRNA2 and sgRNA3 targeting *PvSS*), high editing efficiencies were obtained using PcUbi, while employing the same sgRNAs in combination with Cas9 driven by the 2X35S-Ω promoter resulted in significantly lower gene editing efficiencies, indicating that the 2X35S-Ω promoter may not be consistently reliable. These results demonstrate the potential of employing a hairy root transformation system as a valuable and efficient approach for evaluating the effectiveness of sgRNAs and the impact of different promoters on the editing efficiency. This system holds great promise in enhancing the success rate of CRISPR/Cas9-mediated stable transformation. Alternative methods have been developed for different plant species, primarily relying on transient expression to assess the efficacy of CRISPR/Cas9 editing. Notably, leaf infiltration has been successfully implemented in *A. thaliana* and *N. benthamiana* (Ordon et al., 2017). In the case of wheat, researchers have utilized a protoplast transfection assay and particle bombardment has been used in Barley (Arndell et al., 2019; Michalski et al., 2021).

To evaluate the accuracy of *in silico* prediction tools in forecasting sgRNA efficiency, we compared the predictions from CRISPOR, CRISPR RGEN, and inDelphi with the *in vivo* results. Generally, predicting sgRNA efficiency accurately appears to be challenging. No correlation was observed between the different *in silico* scores for a single sgRNA, nor between the *in vivo* efficiencies and most scores. For instance, sgRNA2 targeting *PvRS1* performed well *in vivo* despite having only moderate *in silico* scores. Conversely, sgRNA2 targeting *PvRS2* exhibited low efficiency in hairy roots but received high *in silico* scores. Nonetheless, the majority of designed sgRNAs demonstrated high *in planta* editing efficiency, which partially aligns with the high Lindel out-of-frame scores used for their initial selection. Moreover, the Lindel repair model accurately predicted indels for all nine sgRNAs, suggesting that the Lindel model can effectively predict gene editing qualities of sgRNAs in common bean. However, sgRNA2 targeting *PvRS2* obtained a high Lindel out-of-frame score of 77 but did not perform as effectively *in planta*, with KO scores ranging from 11.3% (SE = ± 2.9) to 31.7% (SE = ± 5.7). This underscores the importance of conducting an initial sgRNA efficiency screen using our hairy root transformation system to identify efficient sgRNAs despite their predicted efficiencies *in silico*.

### 4.3 Analysis of mutation types and assessing the reliability of *in silico* prediction tools

In our study we detected three times more deletions than insertions, which mirrors the indel distribution observed by Chen et al. (2019). CRISPR/Cas9 activity can result in targeted breaks in plant DNA at specific locations and plants have developed different repair mechanisms to fix the resulting DSBs. However, depending on the repair system used, different types of indels could arise at these locations. C-NHEJ is considered the most prominent repair mechanism within plants, which can result in insertions and deletions of a few base pairs (Puchta, 2005; Weterings and Chen, 2008). Within our hairy root sample pool, the most common mutations were indeed small insertions (1 or 2 bp) and deletions (1 to 5 bp) which are likely the result of C-NHEJ. Especially 1 and 2 bp insertions and deletions were present at high frequencies, an outcome that was also observed in other studies (Ordon et al., 2017; Chen et al., 2019; Zhang et al., 2020; Molla et al., 2022). While it was initially believed that SpCas9 generated blunt ends, an increasing number of studies now suggest that it can also produce 5’ staggered ends (Molla and Yang, 2019). Shou et al. (2018) demonstrated that the HNH nuclease domain of SpCas9 accurately cleaves DNA at the -3 position upstream of the PAM, while the RuvC domain exhibits greater flexibility and can cut even further upstream, generating 1-3 nt overhangs (Shou et al., 2018; Molla and Yang, 2019). These overhangs can be subsequently filled by DNA polymerase and ligated, resulting in small insertions corresponding with the template. Consequently, this leads to predictable insertions, as opposed to the unpredictable outcomes associated with blunt ends (Shou et al., 2018; Molla and Yang, 2019). Our findings are consistent with this hypothesis, as the majority of observed single nucleotide insertions matched with the expected template at the -4 position distal from the PAM. Although the inDelphi algorithm was optimized for human and mouse cell lines, it accurately predicted the insertions and most of the deletions, suggesting that the repair system may be more universally applicable than previously assumed (Shen et al., 2018). The only exception was sgRNA3 targeting *PvRS1*, where the observed *in vivo* outcome was not accurately predicted. However, this sgRNA contained an SNP immediately adjacent to the PAM site, which could have influenced the outcome and therefore should be interpreted with caution. A recent report on rice has also shown that NHEJ-mediated single nucleotide insertions can indeed be predicted based on the DNA sequence at the target loci (Molla et al., 2022). We present the first evidence that this is also applicable to common bean. These results offer promising implications for enhancing precision editing in plants, as they indicate the possibility of predicting NHEJ repair outcomes.

When micro-homologous sequences are found near the DSB, MMEJ can also lead to small deletions. Target sites with high microhomology strength typically possess local microhomologies that are lengthy, GC-rich, or situated near the cut site (Shen et al., 2018). Consequently, repair outcomes are more likely to involve microhomology-based deletions. Upon analyzing the sequences flanking the cleavage site, inDelphi indicated that six of the nine target sites exhibited an average microhomology strength, while two targets demonstrated high microhomology strength. This suggests that some of the observed longer deletions could be attributed to MMEJ in these cases. However, inDelphi suggested that the flanking region of the cleavage site of sgRNA2 targeting *PvRS2* had a high microhomology strength, yet only small deletions were observed in the majority of analyzed hairy roots indicating that the predicted microhomology strength is not always a reliable parameter.

Since common bean is diploid, CRISPR/Cas9 can result in a heterozygous mutation, a biallelic mutation, or a homozygous mutation. When looking at individual hairy roots, the presence of one or two dominant indels (occurrence > 20%) was indeed observed. Additionally, hairy roots may not originate from a single cell as observed in both our and other studies, resulting in a chimeric nature of the root, which could also influence the interpretation of the observed mutations (Limpens et al., 2004; Garagounis et al., 2020). In our study, the number of dominant indels per hairy root varied depending on the sgRNA used and ranged between one and four supporting the hypothesis of the chimeric nature of each hairy root. This observation could potentially also explain the difference in indels observed by the computational programs and *in vivo*. Both computational programs, inDelphi and Lindel, successfully predicted the majority of observed indels, whereas the Bae repair model was less reliable in this regard, suggesting that the model may not be suitable for use in plant systems. Although these models are trained on mutational events in human and mouse cell lines, the Lindel repair model and inDelphi algorithm appear to be valuable for predicting DSB repair outcomes in plant systems as well, particularly for single nucleotide insertions. Additionally, the Lindel model seems to be the most effective tool for predicting sgRNA efficiency in common bean. These results offer promising implications for enhancing precise editing in plants.

## Data availability statement

The original contributions presented in the study are included in the article/**Supplementary Material**. Further inquiries can be directed to the corresponding author.

## Author contributions

Conceptualization, RdK and GA. Methodology, RdK, HD and GA. Software, RdK and RK. Validation, RdK, HD, JG, MT and GA. Formal analysis, RdK, HD and JG. Investigation, RdK HD, JG, RK, MT and GA. Resources, GA. Writing original draft preparation, RdK. Writing review and editing, RdK, HD, JG, MT and GA. Visualization, RdK. Supervision, RdK and GA. All authors contributed to the article and approved the submitted version.
