# Supplementary material for: Fine-tuning CRISPR/Cas9 gene editing in common bean (Phaseolus vulgaris L.) using a hairy root transformation system and in silico prediction models
Source: Front Plant Sci. 2023 Oct 20;14:1233418. doi: 10.3389/fpls.2023.1233418 (PMC10623320; doi:10.3389/fpls.2023.1233418)
Supplement: Supplementary file 1 [file DataSheet_1.docx]

Supplementary Material

Fine-tuning CRISPR/Cas9 gene editing in common bean (*Phaseolus vulgaris* L.) using a hairy root transformation system and *in silico* prediction models

Ramon de Koning, Hana Daryanavard , Joyce Garmyn, Raphaël Kiekens, Mary Esther Muyoka Toili, Geert Angenon^1*^

*** Correspondence:**Geert Angenon
Geert.Angenon@vub.be

# Supplementary Data

**Supplementary Table 1**. Overview of the sgRNAs targeting the RFO biosynthetic genes of common bean.

| Name sgRNA | Target gene | Target exon | Nucleotide sequence | PAM |
| --- | --- | --- | --- | --- |
| sgRNA1_RS1 | *Raffinose synthase 1* (*PvRS1*) | 1 | ^5’^GTTGCCCTAAGGGTGCCACA^3’^ | TGG |
| sgRNA2_RS1 | *Raffinose synthase 1* (*PvRS1*) | 1 | ^5’^GTTGCAACCGGTCCAGAGAG^3’^ | TGG |
| sgRNA3_RS1 | *Raffinose synthase 1* (*PvRS1*) | 2 | ^5’^GGTTCGTGCTGATCGACGAC^3’^ | GGG |
| sgRNA1_RS2 | *Raffinose synthase 2* (*PvRS2*) | 1 | ^5’^GGACTTCCGTCAGAAAAGGG^3’^ | TGG |
| sgRNA2_RS2 | *Raffinose synthase 2* (*PvRS2*) | 2 | ^5’^GTGGAGGGAGGGTGCCCTCC^3’^ | GGG |
| sgRNA3_RS2 | *Raffinose synthase 2* (*PvRS2*) | 2 | ^5’^GGATCCCGTAACGGACCAAG^3^ | AGG |
| sgRNA1_SS | *Stachyose synthase* (*PvSS*) | 1 | ^5’^GTGGGGAACAGATGACTACC^3’^ | AGG |
| sgRNA2_SS | *Stachyose synthase* (*PvSS*) | 3 | ^5’^GGGACCCAATGGGAGTGTTC^3’^ | TGG |
| sgRNA3_SS | *Stachyose synthase* (*PvSS*) | 4 | ^5’^GCAGTACCTCCGCCTGATTG^3’^ | AGG |

**Supplementary Table 2**. Primers used for diagnostic PCR of pEn-C1.1.

| Name | Forward primer nucleotide sequence (nt) | Tm (°C) |  |
| --- | --- | --- | --- |
| SS42 | ^5’^TCCCAGGATTAGAATGATTAGG^3’^ | 51.2 |  |
| Name | Reverse primer nucleotide sequence (nt) | Tm (°C) | Amplicon length (bp) |
| RV_sgRNA1-RS1 | ^5’^TGTGGCACCCTTAGGGCAAC^3’^ | 59.7 | 279 |
| RV_sgRNA2-RS1 | ^5’^CTCTCTGGACCGGTTGCAAC^3’^ | 58 | 279 |
| RV_sgRNA3-RS1 | ^5’^GTCGTCGATCAGCACGAACC^3’^ | 58.6 | 279 |
| RV_sgRNA1-RS2 | ^5’^ATCCGCCACAGAGCGCATGC^3’^ | 63.3 | 279 |
| RV_sgRNA2-RS2 | ^5’^CAATCAGGCGGAGGTACTGC^3’^ | 57.9 | 279 |
| RV_sgRNA3-RS2 | ^5’^GAACACTCCCATTGGGTCCC^3’^ | 57.3 | 279 |
| RV_sgRNA1-SS | ^5’^GGTAGTCATCTGTTCCCCA^3’^ | 52.9 | 279 |
| RV_sgRNA2-SS | ^5’^GAACACTCCCATTGGGTCC^3’^ | 55 | 279 |
| RV_sgRNA3-SS | ^5’^CAATCAGGCGGAGGTACTG^3’^ | 54.9 | 279 |

**Supplementary Table 3**. Primers used for diagnostic PCR of pMR356-GFP and pMR394-GFP with sgRNA.

| Forward primer name | Nucleotide sequence (nt) | Tm (°C) | Amplicon length (bp) |
| --- | --- | --- | --- |
| Reverse primer name |  |  |  |
| Cas9v3 | ^5’^GGCTTGTTGTGTTATGAATTTGTGG ^3’^ | 56.3 | 1095 |
| OLEv2 | ^5’^CAGAATGTGGCGTTGGCATC ^3’^ | 57.3 |  |
| Cas9V3 | ^5’^GGCTTGTTGTGTTATGAATTTGTGG ^3’^ | 56.3 | 634 |
| sgRNA_RV | ^depends on sgRNA^ |  |  |
| Cas9_1_F | ^5’^CTGAGGAAACCATCACCCCT ^3’^ | 56.7 | 857 |
| Cas9_1_R | ^5’^ATGCCTTCCCATCACCTTCA ^3’^ | 56.5 |  |
| PVS1_FW | ^5’^ TTGGGAACCGGTCACACAT ^3’^ | 56.3 | 2396 |
| SmR_RV | ^5’^ GTGATCGCCGAAGTATCGACT ^3’^ | 57 |  |
| RB_FW | ^5’^ TGGCGGGTAAACCTAAGAGA ^3’^ | 55.1 | 3445 |
| BLANK_RV | ^5’^ ACATTAGGAACCGGCGGATG ^3’^ | 57.2 |  |
| Kanr_FW | ^5’^ GGTGCCCTGAATGAACTCCA ^3’^ | 57.4 | 4187 (pMR356-GFP) & 4063 (pMR394-GFP) |
| Cas9_1_R | ^5’^ ATGCCTTCCCATCACCTTCA ^3’^ | 56.5 |  |
| eGFP_3 | ^5’^ ACGTAAACGGCCACAAGTTC ^3’^ | 56.2 | 546 |
| eGFP_5 | ^5’^ TGCTCAGGTAGTGGTTGTCG ^3’^ | 56.8 |  |

**Supplementary Table 4.** Sequencing primers to amplify the different target regions of the RFO biosynthetic genes of common bean.

| Name sgRNA | Target gene | Forward primer name | Nucleotide sequence (nt) | Tm (°C) | Amplicon length (bp) |
| --- | --- | --- | --- | --- | --- |
|  |  | Reverse primer name |  |  |  |
| sgRNA1_RS1 | *Raffinose synthase 1* (*PvRS1*) | RS1gRNA1-2_SEQ_FW | ^5’^TGGCTTCAACC  CTTCACCTT^3’^ | 56.9 | 451 |
|  |  | RS1gRNA1-2_SEQ_RV | ^5’^TCATTAGGGG  TGCAGAAAGC^3’^ | 55.1 |  |
| sgRNA2_RS1 | *Raffinose synthase 1* (*PvRS1*) | RS1gRNA1-2_SEQ_FW | ^5’^TGGCTTCAACC  CTTCACCTT^3’^ | 56.9 | 451 |
|  |  | RS1gRNA1-2_SEQ_RV | ^5’^TCATTAGGGGT  GCAGAAAGC^3’^ | 55.1 |  |
| sgRNA3_RS1 | *Raffinose synthase 1* (*PvRS1*) | RS1gRNA3_SEQ_FW | ^5’^GAGAGTGTGG  TGTACGTGCA^3’^ | 57.1 | 552 |
|  |  | RS1gRNA3_SEQ_RV | ^5’^CGTCATCTTCA  ACCCCTCCG^3’^ | 57.8 |  |
| sgRNA1_RS2 | *Raffinose synthase 2* (*PvRS2*) | RS2gRNA1_SEQ_FW | ^5’^ATATAAACCC  ACCAACCCAC^3’^ | 52.3 | 415 |
|  |  | RS2gRNA1_SEQ_RV | ^5’^CAGCGAGTCAT  TCTTGTCGAG^3’^ | 55.6 |  |
| sgRNA2_RS2 | *Raffinose synthase 2* (*PvRS2*) | RS2gRNA2_SEQ_FW | ^5’^ACCGCGCCAG  GTATATTAAA^3’^ | 54 | 420 |
|  |  | RS2gRNA2_SEQ_RV | ^5’^CGCTCCTAAA  CCCCTCCTTC^3’^ | 57.4 |  |
| sgRNA3_RS2 | *Raffinose synthase 2* (*PvRS2*) | RS2gRNA3_SEQ_FW | ^5’^AGAAGTTTGGT  TGGTGTACGTG^3’^ | 55.4 | 383 |
|  |  | RS2gRNA3_SEQ_RV | ^5’^GAACCTCAGG  TCTGACACCA^3’^ | 56.4 |  |
| sgRNA1_SS | *Stachyose synthase* (*PvSS*) | SSgRNA1_SEQ_FW | ^5’^GATTTTAGTGA  GGGTGGCGTG^3’^ | 56 | 405 |
|  |  | SSgRNA1_SEQ_RV | ^5’^ACACCCTCCA  CTTTCGTTGC^3’^ | 58 |  |
| sgRNA2_SS | *Stachyose synthase* (*PvSS*) | SSgRNA2_SEQ_FW | ^5’^TCACTTCTGAGGA  ATTTTATTGTGC^3’^ | 53.8 | 394 |
|  |  | SSgRNA2_SEQ_RV | ^5’^AGGCAGTCTCT  TGTTGGAAGG^3’^ | 57 |  |
| sgRNA3_SS | *Stachyose synthase* (*PvSS*) | SSgRNA3_SEQ_FW | ^5’^ACCACACAATTGG  TATCAATCTAAC^3’^ | 53.4 | 462 |
|  |  | SSgRNA3_SEQ_RV | ^5’^GTCTCCCACCA  CCCTTAACC^3’^ | *57.5* |  |

**Supplementary Table 5.** Summary of the sgRNA efficiencies to induce insertions and deletions (INDEL score) and out-of-frame or knock-out mutations (KO score) in the target genes of *P. vulgaris* cv. CIAP7247F. Scores are shown with the standard error of the mean.

|  |  | **pMR356-GFP** | |  | **pMR394-GFP** | | |
| --- | --- | --- | --- | --- | --- | --- | --- |
| **Target** | **sgRNA** | **n** | **INDEL score** | **KO score** | **n** | **INDEL score** | **KO score** |
| *PvRS1* | 1 | 16 | 0% (±0.0) | 0% (±0.0) | 15 | 68.2% (±6.2) | 49.0% (±9.6) |
|  | 2 | 15 | 75.1% (±4.3) | 55.3% (±8.3) | 16 | 84.8% (±2.4) | 73.8% (±6.9) |
|  | 3 | 15 | 2.2% (±1.2) | 2.2% (±1.2) | 18 | 0.3% (±0.27) | 0.3% (±0.27) |
| *PvRS2* | 1 | 15 | 77.6% (±2.8) | 65.4% (±4.4) | 17 | 32.7% (±9.8) | 32.7% (±9.8) |
|  | 2 | 17 | 11.3% (±2.9) | 11.3% (±2.9) | 15 | 32.8 (±6.0) | 31.7 (±5.7) |
|  | 3 | 15 | 77.6% (±4.2) | 68.7% (±6.2) | 15 | 2.7% (±0.7) | 2.7% (±0.7) |
| *PvSS* | 1 | 17 | 81.0% (±1.5) | 50.9% (±7.5) | 16 | 80.9% (±6.3) | 75.9% (±8.0) |
|  | 2 | 17 | 71.0% (±5.3) | 57.4% (±5.8) | 15 | 3.2% (±0.9) | 2.7% (±0.9) |
|  | 3 | 15 | 84.7% (±2.6) | 65.5% (±6.6) | 19 | 0.9% (±0.5) | 0.7% (±0.4) |

**Supplementary Table 6.** Sequences of the 2X35S-Ω and PcUbi and *A.thaliana* U6-26 promoters.

| *A. thaliana*  U6-26 | ^5'^CTTTTTTTCTTCTTCTTCGTTCATACAGTTTTTTTTTGTTTATCAGCTTACATTTTCTTGAACCGTAGCTTTCGTTTTCTTCTTTTTAACTTTCCATTCGGAGTTTTTGTATCTTGTTTCATAGTTTGTCCCAGGATTA^3’^ |
| --- | --- |
| 2X35S-Ω | ^5'^CAACATGGTGGAGCACGACACTCTGGTCTACTCCAAAAATGTCAAAGATACAGTCTCAGAAGATCAAAGGGCTATTGAGACTTTTCAACAAAGGATAATTTCGGGAAACCTCCTCGGATTCCATTGCCCAGCTATCTGTCACTTCATCGAAAGGACAGTAGAAAAGGAAGGTGGCTCCTACAAATGCCATCATTGCGATAAAGGAAAGGCTATCATTCAAGATCTCTCTGCCGACAGTGGTCCCAAAGATGGACCCCCACCCACGAGGAGCATCGTGGAAAAAGAAGAGGTTCCAACCACGTCTACAAAGCAAGTGGATTGATGTGATAACATGGTGGAGCACGACACTCTGGTCTACTCCAAAAATGTCAAAGATACAGTCTCAGAAGATCAAAGGGCTATTGAGACTTTTCAACAAAGGATAATTTCGGGAAACCTCCTCGGATTCCATTGCCCAGCTATCTGTCACTTCATCGAAAGGACAGTAGAAAAGGAAGGTGGCTCCTACAAATGCCATCATTGCGATAAAGGAAAGGCTATCATTCAAGATCTCTCTGCCGACAGTGGTCCCAAAGATGGACCCCCACCCACGAGGAGCATCGTGGAAAAAGAAGAGGTTCCAACCACGTCTACAAAGCAAGTGGATTGATGTGACATCTCCACTGACGTAAGGGATGACGCACAATCCCACTATCCTTCGCAAGACCCTTCCTCTATATAAGGAAGTTCATTTCAATTTGGAGAGGACACGCTCGAGTATAAGAGCTCATTTTTACAACAATTACCAACAACAACAAACAACAAACAACATTACAATTACATTTACAATTATCGATACA^3'^ |
| PcUbi | ^5’^AAAAATTACGGATATGAATATAGGCATATCCGTATCCGAATTATCCGTTTGACAGCTAGCAACGATTGTACAATTGCTTCTTTAAAAAAGGAAGAAAGAAAGAAAGAAAAGAATCAACATCAGCGTTAACAAACGGCCCCGTTACGGCCCAAACGGTCATATAGAGTAACGGCGTTAAGCGTTGAAAGACTCCTATCGAAATACGTAACCGCAAACGTGTCATAGTCAGATCCCCTCTTCCTTCACCGCCTCAAACACAAAAATAATCTTCTACAGCCTATATATACAACCCCCCCTTCTATCTCTCCTTTCTCACAATTCATCATCTTTCTTTCTCTACCCCCAATTTTAAGAAATCCTCTCTTCTCCTCTTCATTTTCAAGGTAAATCTCTCTCTCTCTCTCTCTCTCTGTTATTCCTTGTTTTAATTAGGTATGTATTATTGCTAGTTTGTTAATCTGCTTATCTTATGTATGCCTTATGTGAATATCTTTATCTTGTTCATCTCATCCGTTTAGAAGCTATAAATTTGTTGATTTGACTGTGTATCTACACGTGGTTATGTTTATATCTAATCAGATATGAATTTCTTCATATTGTTGCGTTTGTGTGTACCAATCCGAAATCGTTGATTTTTTTCATTTAATCGTGTAGCTAATTGTACGTATACATATGGATCTACGTATCAATTGTTCATCTGTTTGTGTTTGTATGTATACAGATCTGAAAACATCACTTCTCTCATCTGATTGTGTTGTTACATACATAGATATAGATCTGTTATATCATTTTTTTTATTAATTGTGTATATATATATGTGCATAGATCTGGATTACATGATTGTGATTATTTACATGATTTTGTTATTTACGTATGTATATATGTAGATCTGGACTTTTTGGAGTTGTTGACTTGATTGTATTTGTGTGTGTATATGTGTGTTCTGATCTTGATATGTTATGTATGTGCAGC^3’^ |


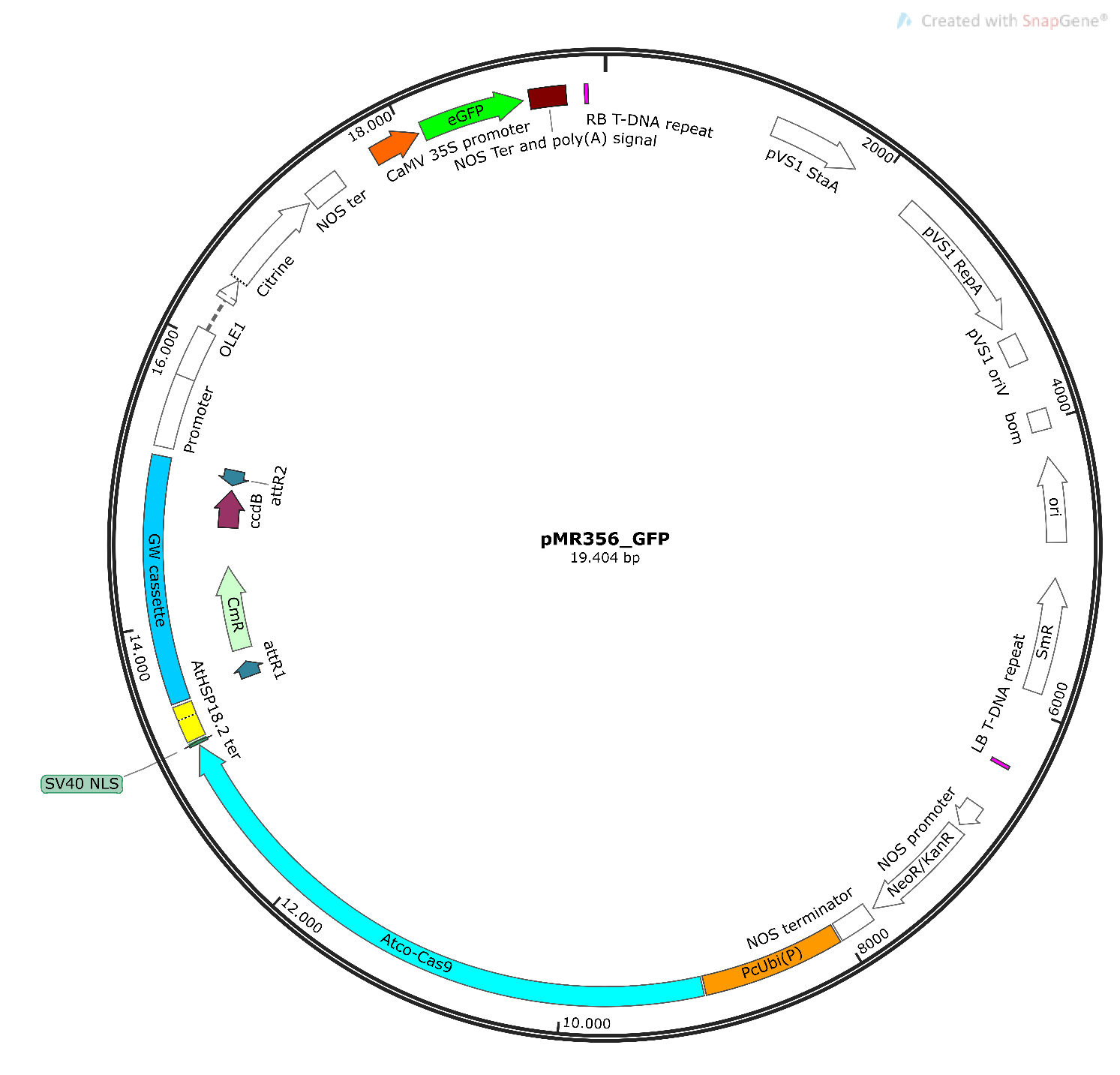


**Supplementary Figure 1.** Vector map of pMR356-GFP.


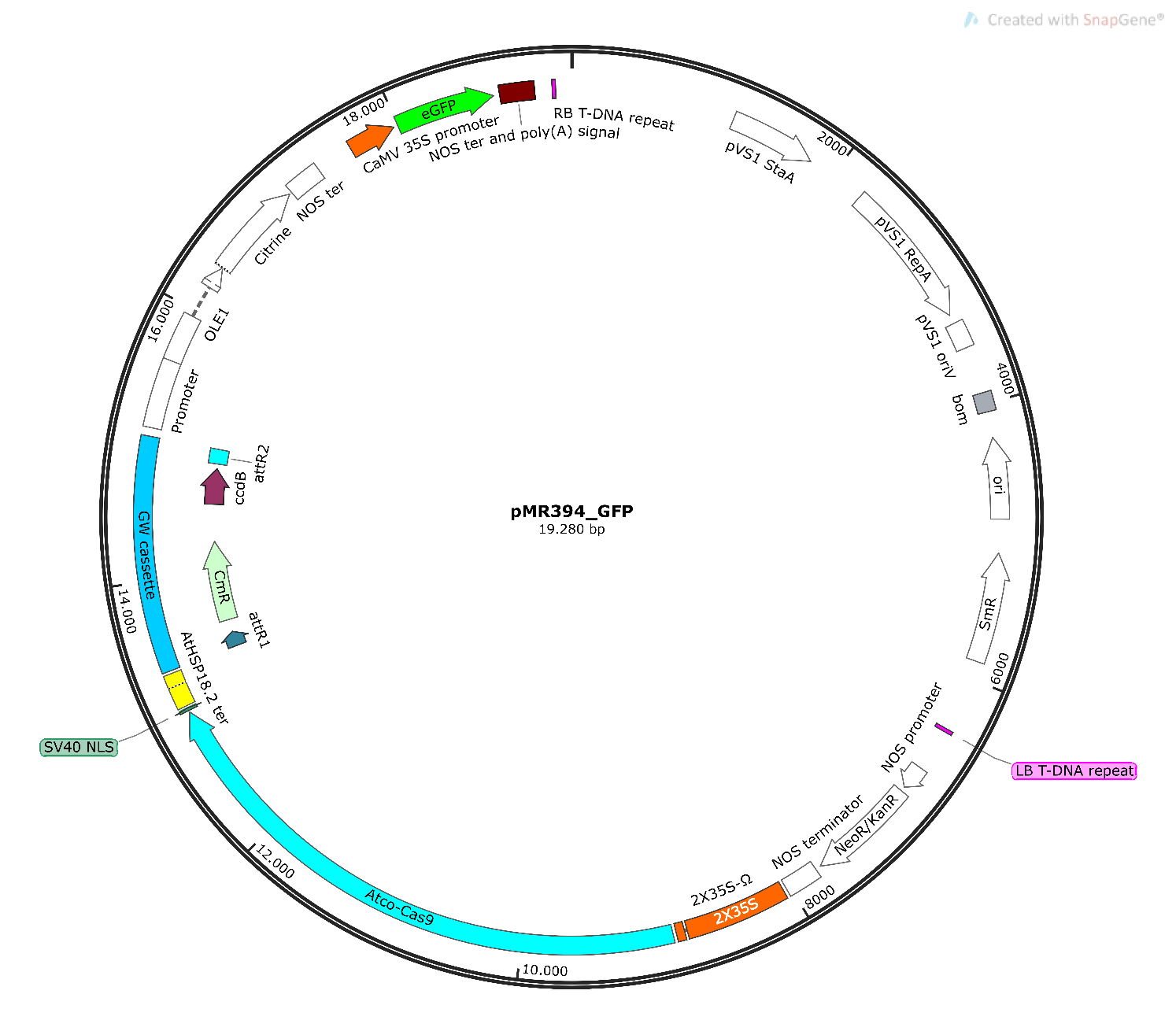


**Supplementary Figure 2.** Vector map of pMR394-GFP.
